# Albumin Fusion of Interleukin-28B: Production and Characterization of Its Biological Activities and Protein Stability

**DOI:** 10.1371/journal.pone.0064301

**Published:** 2013-05-31

**Authors:** Jin Zhao, Youhui Si, Min Cheng, Yang Yang, Yuqiang Niu, Xiang Li, Xiuying Liu, Wei Yang

**Affiliations:** MOH Key Laboratory of Systems Biology of Pathogens, Institute of Pathogen Biology, Chinese Academy of Medical Sciences & Peking Union Medical College, Beijing, China; University of California, Merced, United States of America

## Abstract

The cytokine interleukin-28B (IL-28B) has potential antiviral properties and regulatory roles in adaptive cellular immunity. A genome-wide association study identified a single nucleotide polymorphism near the IL-28B gene that strongly predicts response to hepatitis C treatment with interferon and ribavirin. In this study, we produced human serum albumin (HSA) fused to interleukin-28B (HSA-IL28B) in an attempt to determine the effects of albumin fusion on anti-Hepatitis C virus (HCV) activity and protein stability. HSA-IL28B was expressed at high levels in the yeast expression system we used and was easily purified. The biological activities of IL-28B were only retained when HSA was fused at the N-terminus. Compared with the native IL-28B, HSA-IL28B showed improved protein stability. HSA-IL28B inhibited HCV infection through the membrane receptors IL28R1and IL10R2. Additionally, we demonstrated that HSA-IL28B was able to induce interferon-stimulated genes, phosphorylate intracellular STAT1, and act in restricted cell types. Our findings highlight the potential clinical applications of the fusion protein during virus infection and for immune regulation.

## Introduction

Interferons (IFNs) are cytokines produced naturally, or upon pathogen challenge. Currently, three types of IFNs (types I, II and III) have been characterized, with type I IFN widely used to treat hepatitis C, leukemia, lymphomas, and recurrent melanoma. Hepatitis C virus (HCV) infects an estimated 170 million people worldwide [1], resulting in high rates of chronic infection and increasing the risk for severe liver diseases. Co-infection of HCV with human immunodeficiency virus (HIV), and other human pathogens, is a massive challenge facing health authorities and will require the development of innovative therapeutic strategies to combat it. The current standard treatment regimen for chronic hepatitis C is a combination of type I IFN and ribavirin therapy [2]. Although direct antiviral agents (DAAs) targeting HCV NS3/4A protease were recently approved by the Food and Drug Administration, existing and adaptive mutations conferring drug resistance have forced the development of more novel anti-HCV therapeutics.

Interleukin-28 (IL-28) has two isoforms, IL-28A and IL-28B, and are a part of the type III IFN family comprising IL-29, IL-28A and IL-28B (also known as IFN-λ1, -λ2, and -λ3, respectively). Recent genome-wide association studies have demonstrated that a genetic polymorphism in the il-28b gene was strongly associated with a sustained virological response during IFN treatment of chronic hepatitis C patients [3], [4], [5]. Using a chimpanzee model and primary human hepatocyte cultures, Park and colleagues showed that HCV infection stimulated strong type III but weak type I IFN responses in the liver and plasma [6], [7], [8]. The biological significance of this kind of IL28B induction upon clearance of HCV remains largely unknown.

The type III IFNs transduce signals by binding to and stimulating a heterodimeric membrane receptor. This receptor is composed of a long, specific IL28 α chain (IL28R1) and a short, widely distributed IL10 β chain (IL10R2). Similar to type I IFNs, activation of IFN-λ receptors leads to phosphorylation of the Janus tyrosine kinase-signal transducer and activator of the transcription (JAK-STAT) pathway. Furthermore, phosphorylated STAT1 and STAT2, together with IFN regulatory factor 9 (IRF-9), form the IFN-stimulated gene factor 3 (ISGF3) complex. This regulates typical IFN-induced genes such as OAS and MxA. The therapeutic potential of type III IFNs to viral infection has been documented *in vitro* and *in vivo* for HCV, HIV, hepatitis B virus, herpes simplex virus and West Nile virus. Additionally, accumulating evidence suggests that type III IFNs have specific effects on the regulation of the immune system and inhibition of tumor cell growth.

Type III IFNs were first used in a clinical setting to treat hepatitis C. A pegylated IL-29 has been developed and is being used in phase 2 clinical trials. This particular molecule has shown some promising outcomes compared with pegylated IFN-α, with better tolerance and lower adverse effects observed for pegylated IL-29. Based on these results, we believe that type III IFNs can be applied as novel treatments for chronic hepatitis C. All type III IFNs were compared, and it was shown that IL-28B had potent antiviral activity, along with IL-29 and IL-28A [9]. We previously prepared a recombinant IL28B protein that performed well against HCV and restricted cell-type responsiveness *in vitro*. However, development of an IL-28B molecule with sustained long-term effects has not been reported, but is urgently needed for treatment of HCV infections.

Several technologies have been developed to enhance the pharmacokinetics of polypeptides, including chemical/covalent modification, micro-encapsulation and the use of protease-resistant variants. New approaches to improving pharmacokinetics of small proteins are based on binding to, or fusion with, circulating serum proteins such as human serum albumin (HSA). Albumin is the most abundant protein in the blood plasma; it is produced in the liver as a monomeric protein of 67 kDa [10]. Besides its role in regulating the osmotic pressure of plasma, its physiologic functions include the transport of metabolites such as long-chain fatty acids, bilirubin, steroid hormones, tryptophan, and calcium. Albumin also binds with high affinity to a broad range of drugs, thereby influencing their pharmacokinetic properties [11]. Albumin has a simple molecular structure and is highly stable, is abundantly present in vascular and extravascular compartments, and has a circulation half-life of 19 days in humans. HSA has been used as a macromolecular carrier for drug delivery. It has also been successfully used to generate fusion proteins with hormones (insulin, human growth hormone) [12], [13] and cytokines (interferon-α, interferon-β, IL-2) [14]. This has resulted in reduced immunogenicity and modulation of pharmacokinetic properties, thus improving the therapeutic efficacy of these molecules.

In this study, we expressed and purified IL-28B that was fused to HSA (HSA-IL28B), and evaluated its efficacy against HCV.

## Materials and Methods

### Yeast Strains, Cells and Reagents

The PichiaPink™ Secreted Protein Expression Kit (Cat. No. A11151) and PichiaPink™ Media Kit (Cat. No. A11156) were purchased from Invitrogen (Carlsbad, CA, USA). The human hepatoma cell line, Huh7.5.1, was a gift from Dr. Francis V. Chisari (Scripps Research Institute, La Jolla, CA, USA). The Huh7 cell line was from Apath Inc. HepG2, Caco-2, HeLa, HEK293T, A549, DU145, K562, MCF7 and U251 cells were obtained from ATCC (Manassas, VA, USA). The HCV genotype 1b replicon-containing cell line (2−3+) was kindly provided by Dr. Stanley Lemon (University of Texas Medical Branch, Galveston, TX, USA). T4 DNA ligase and all restriction enzymes were purchased from New England Biolabs (Ipswich, MA, USA). SP-Sepharose Fast Flow was from GE Healthcare (Piscataway, NJ, USA). PEG-IFNα2b was from Schering Plough (Kenilworth, NJ, USA) and ribavirin was obtained from the Beijing YouAn Hospital. Recombinant IL28B protein was purchased from R&D Systems (Minneapolis, MN, USA). Antibodies against STAT1, MX1, and ISG15 were obtained from Abcam, Cell Signaling Technology (phospho-STAT1-Tyr701), Thermo Scientific (HCV Core), and Sigma (β-actin). Horseradish peroxidase (HRP)-conjugated secondary antibodies were purchased from Jackson ImmunoResearch (West Grove, PA, USA).

### Expression and Purification of HSA-IL28B

The human IL28B cDNA sequence (GenBank No. NM_172139) was optimized using *Pichia* codon bias and synthesized by Genscript (Piscataway, NJ, USA). The coding region without a signal peptide (residues 18–196) was fused to the N- or C-terminus of HSA by overlap extension PCR using specific primers (**Table 1**). The fragments fused to the N- or C-terminus were inserted into the yeast expression vector pPinkα-HC, between the *Stu*I and *Kpn*I sites, respectively. After verification by DNA sequencing, the resulting plasmids (pPinkαHC-opt-N-HSA-IL28B and pPinkαHC-opt-IL28B-HSA-C) were linearized with *Spe*I and introduced into PichiaPink Expression Strains by electroporation using a Micropulser (Bio-Rad, Hercules, CA, USA) according to the manufacturer’s instructions. Expression of N-HSA-IL28B and IL28B-HSA-C was performed as previously described [15]. The pPinkα-HC plasmid was also transformed into cells and served as a negative control.

**Table 1:** Primer sequences used in the design and construction of fusion vectors.

Primers	Nucleotide sequence (5′ to 3′ )	Restriction sites
N-HSA-IL28B-F1	5′-GTA TCT CTC GAG AAA AGG CCT GAT GCA CAC AAG AGT GAG-3′	*Stu* I
N-HSA-IL28B-R1	5′-GCA TCC GGG AGA GCC CCG CGT AAG CCT AAG GCA GCT TGA C -3′	/
N-HSA-IL28B-F2	5′-GTC AAG CTG CCT TAG GCT TAC GCG GGG CTC TCC CGG ATG C -3′	/
N-HSA-IL28B-R2	5′-TTT AAA TGG CCG GCC GGT ACC TCA GAC ACA CAG GTC CCC G-3′	*Kpn* I
IL28B-HSA-C-F1	5′-GTA TCT CTC GAG AAA AGG CCT CGC GGG GCT CTC CCG GAT G-3′	*Stu* I
IL28B-HSA-C-R1	5′-CCT CAC TCT TGT GTG CAT CGA CAC ACA GGT CCC CGC TGG-3′	/
IL28B-HSA-C-F2	5′-CCA GCG GGG ACC TGT GTG TCG ATG CAC ACA AGA GTG AGG-3′	/
IL28B-HSA-C-R2	5′-TTT AAA TGG CCG GCC GGT ACC TTA TAA GCC TAA GGC AGC-3′	*Kpn* I

To achieve high levels of protein expression, methanol concentration (2, 3 and 4%) and induction time (24, 48, and 72 h) were optimized. Expression levels were analyzed by SDS-PAGE. Affi-Gel blue affinity gel (Blue Gel) that was covalently crosslinked with Blue F3GA dye was purchased from Bio-Rad and applied for the affinity purification of fusion proteins. Under optimal expression conditions, supernatant from yeast culture media was harvested by centrifugation and applied to a column containing Blue Gel pre-equilibrated with Buffer A (50 mM HEPES, pH 7.0). After loading, the gel was completely washed with Buffer A and the target protein eluted with Buffer B (50 mM HEPES, 0.3 M NaCl, pH 7.0). The purified samples were analyzed by SDS-PAGE and MALDI-TOF mass spectrometry, then freeze-dried for subsequent biological assays.

### Cell Culture-derived HCV (HCVcc) and Antiviral Assays

The production of firefly luciferase HCV reporter viruses (Jc1-Luc HCVcc) was performed as described elsewhere [15], [16]. Briefly, linearized pFK-Luc-Jc1 plasmid with a T7 promoter was transfected into naive Huh7.5.1 cells with Lipofectimin LTX (Invitrogen). This was followed by infection with a vaccinia virus (VV), which encoded the T7 RNA polymerase, at 8 h post-transfection. Culture supernatants at 48–72 h post-transfection were collected and filtered through 0.1-µm filters (Millipore, Billerica, MA, USA) twice to remove the VV and obtain Jc1-Luc HCVcc. For the antiviral assay, Huh7.5.1 cells infected with Jc1-Luc HCVcc were treated with various concentrations of HSA-IL28B or commercial IL28B. Antiviral effects were evaluated by measuring luciferase activity (Promega, Madison, WI, USA).

### *In vitro* Protease Degradation Assay

To determine whether the HSA-IL28B fusion protein is more resistant to degradation by proteases, *in vitro* protease degradation assay of HSA-IL28B and native IL-28B were performed as described elsewhere [17]. HSA-IL28B or native IL-28B was incubated in 100 µl human serum with or without protease inhibitor cocktail for 3, 9, or 24 hours at 37°C (final concentration 10 ng/ml for HSA-IL28B or 0.625 ng/ml for native IL-28B). Antiviral effects were then determined by measuring luciferase activity as described above.

### Pharmacokinetic Analysis

A single dose pharmacokinetic studies were conducted in CD-1 mice. Briefly, 12 normal CD-1 mice were randomly divided into two groups, each containing six animals. The mice were acclimatized to standard laboratory conditions of 12-h light/12-h dark cycle with free access to rodent chow and water. Mice were administered a single subcutaneous (s.c.) injection of 0.5 mg/kg of HSA-IL28B and native IL-28B. Blood samples were collected at 0, 30 min, 2 h, 6 h, 12 h, 24 h, 48 h, 72 h, 120 h and 144 h. Plasma concentrations of HSA-IL28B and native IL-28B were determined by ELISA.

### Receptor Blocking Assay

Serially diluted antibodies against IL28BR1 or IL10R2 (R&D Systems) were pre-incubated with HCVcc-infected Huh7.5.1 cells at 37°C for 2 h prior to HSA-IL28B (12×10–3 mg/L) treatment. These were removed by washing. A parallel IgG isotype treatment was used as a negative control. The cells were further cultured for 2 days in the presence of HSA-IL28B before luminescence was measured.

### Western Blot Analyses

Western blot assays were performed as previously described [15].

### Statistical Analyses

Data analyses were performed using the t-test for two independent samples. A P-value less than 0.05 was considered statistically significant. All experiments were performed in triplicate, and the values are presented as the mean ± SD.

## Results

### Expression of HSA-IL28B

**Figure 1:**
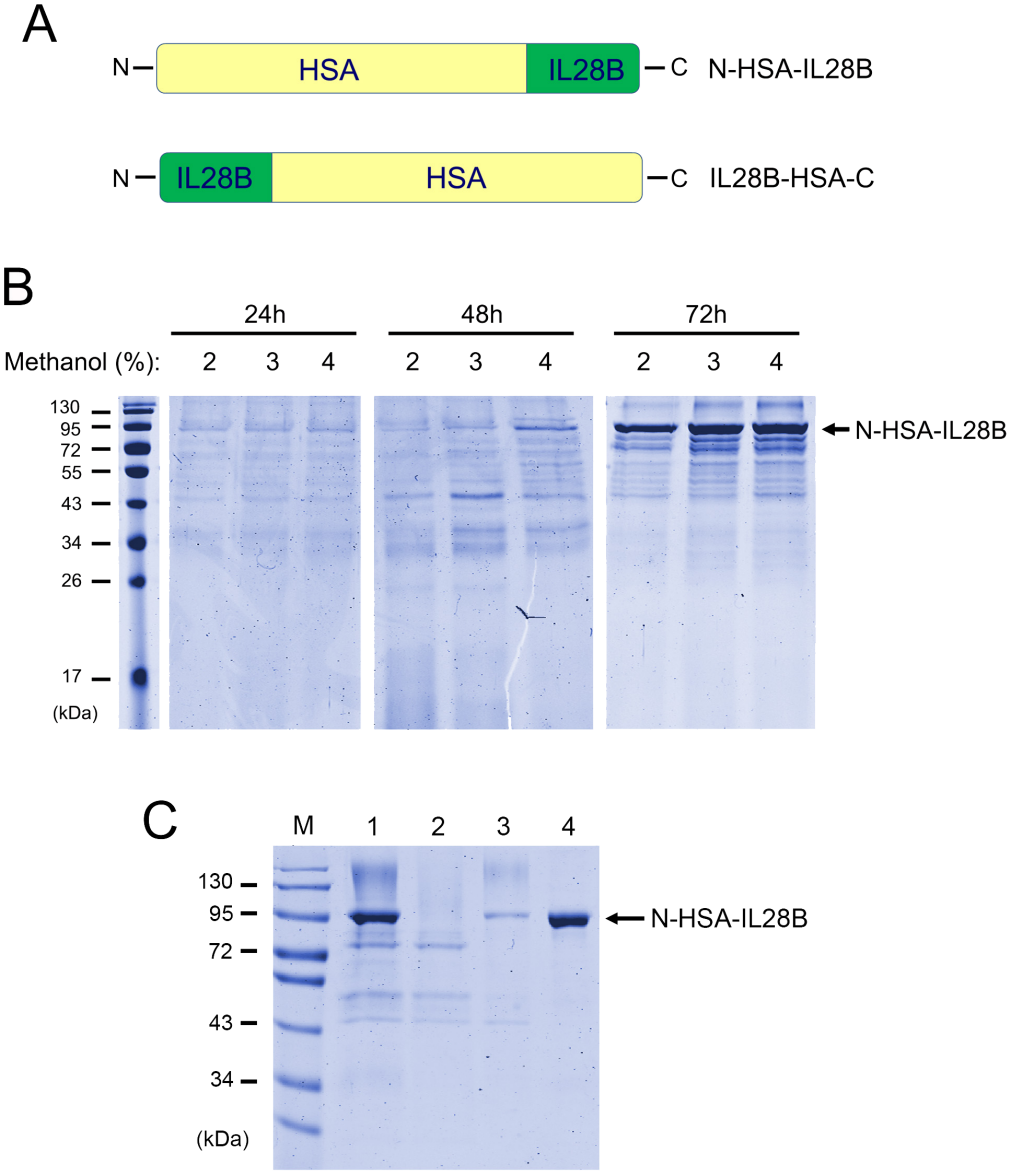
Design and production of HSA-IL28B. (A) Schematic outlining production of the HSA-IL28B fusion protein. HSA was fused at either the N- or C-terminal of IL-28B. (B) Optimization of expression conditions for N-HSA-IL28B. Methanol was supplemented every 24 h to a final concentration of 2, 3 or 4%. Supernatants from yeast cell cultures were collected at 24, 48, and 72 h post-induction and analyzed for recombinant protein production. (C) Purification of N-HSA-IL28B. After methanol induction, N-HSA-IL28B was purified using one-step Blue Gel affinity purification. Lane 1, supernatants; 2, flow-through fraction; 3 and 4, elution fractions with 0.3 M NaCl.

We expressed HSA-IL28B, with the HSA fused at either the N- or C-terminal of IL-28B, and designated it as N-HSA-IL28B or IL28B-HSA-C, respectively (**Fig. 1A**). To obtain a high level of expression, the coding sequence of IL-28B was optimized for *Pichia*. Recombinant plasmids were transformed into electrocompetent PichiaPink yeast cells, and more than 100 clones screened by SDS-PAGE for expression levels of recombinant protein. The yeast clone with the highest expression level of N-HSA-IL28B was selected for further recombinant protein expression optimization. As shown in **Fig. 1B**, fermentation media subject to various induction times and methanol concentrations were analyzed for the expression of N-HSA-IL28B by SDS-PAGE and Coomassie blue staining. Although some differences in the methanol concentration-dependent expression were noticed, a minimum of 72 h was required for induction. Yeast cells secreted a major methanol-dependent polypeptide of the expected size (approximately 95 kDa; **Fig. 1B**). The estimated yield of recombinant N-HSA-IL28B was 80 mg/L, and this was achieved using the shaken bottle method according to the protocol of “PichiaPink™ Expression System” (For High-level and Large-scale Expression and Secretion of Bioactive Recombinant Proteins in *Pichia pastoris*, Invitrogen).

### Purification and Characterization of HSA-IL28B

As shown in **Fig. 1C**, N-HSA-IL28B was captured by Blue Gel, and the eluted protein peak was present at 0.3 M NaCl. Purified N-HSA-IL28B was visible as a single band at approximately 95 kDa, with purity greater than 95% (**Fig. 1C**). The resulting protein product was subjected to MALDI-TOF MS (**Figure S1, S2 and S3**). Recombinant IL28B-HSA-C was purified and characterized in the same way as for N-HSA-IL28B (data not shown).

### Topology of HSA-IL28B Determines anti-HCV Activity

**Figure 2:**
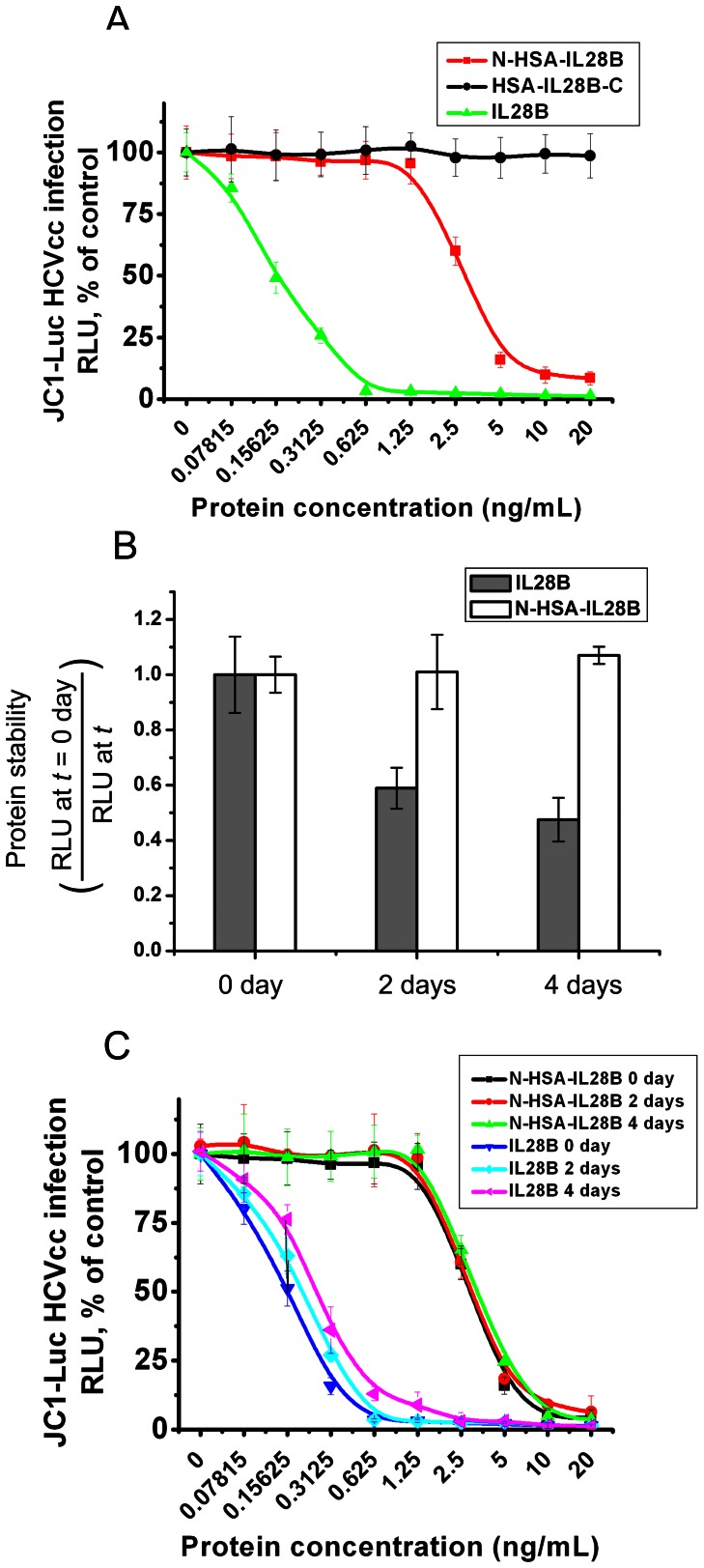
HSA-IL28B demonstrates antiviral activity against HCV and improved protein stability. (A) Huh7.5.1 cells were infected with Jc1-Luc HCVcc one day before treatment with N-HSA-IL28B or IL28B-HSA-C. HCV production was evaluated by measuring intracellular luciferase activity. (B) Native IL-28B or N-HSA-IL28B were incubated with culture media at 37°C for the indicated periods, and their antiviral activities evaluated using Jc1-Luc HCVcc in Huh7.5.1 cells. (C) A serial dilution of Native IL-28B or N-HSA-IL28B were incubated with culture media at 37°C for the indicated periods, and their antiviral activities were evaluated by measuring intracellular luciferase activity.

We determined the anti-HCV activity of both HAS-IL28B forms using Jc1-Luc HCVcc. Fusion of HSA at the N-terminus of IL-28B resulted in a retention of IL-28B antiviral activity (IC50 of 3 ng/mL). However, activity was significantly lower compared with native human IL-28B (IC50 of 0.15 ng/mL) (**Fig. 2A**) [15]. Fusion of HSA at the C-terminus of IL-28B completely abolished its activity for suppressing HCV infection.

### HSA Fusion Improves Stability of Recombinant IL-28B

The antiviral activity of native IL-28B gradually decreased over time in storage, declining to less than 50% activity after 4 days in storage. The activity of HSA-IL28B was completely retained during incubation and storage (**Fig. 2B and 2C**).

### *In vitro* Protease Degradation Assay

**Figure 3:**
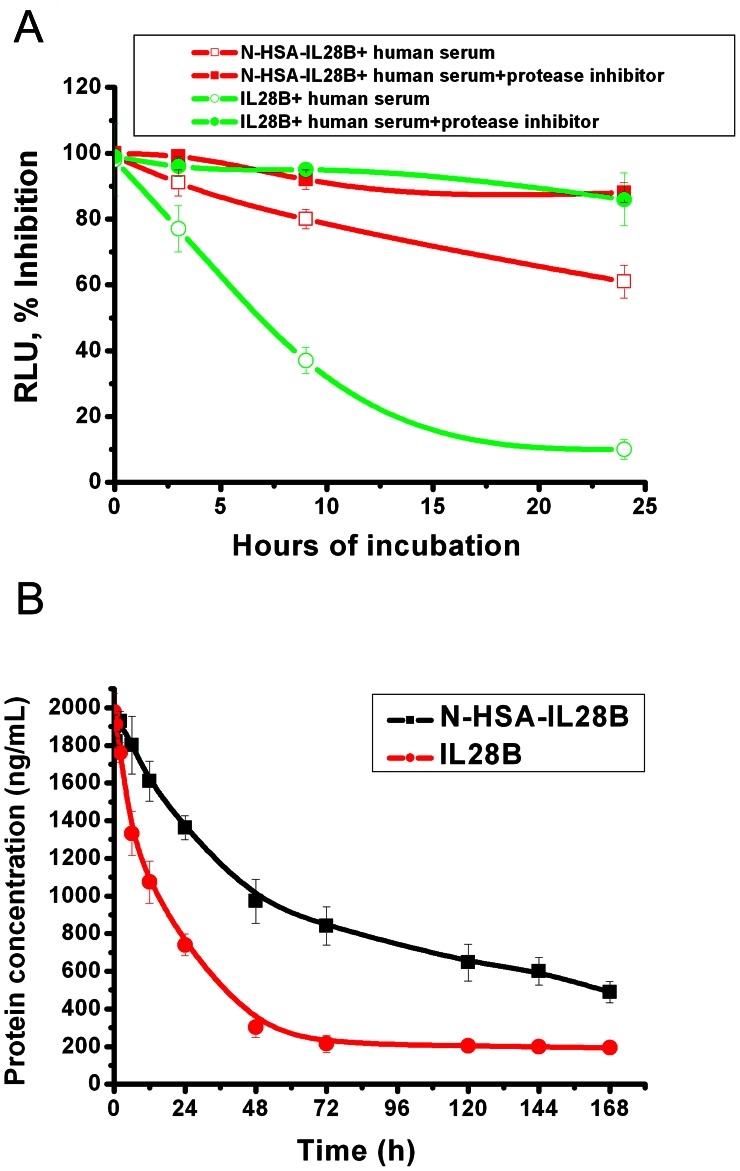
*In vitro* protease degradation assay and Pharmacokinetics analysis. (A) HSA-IL28B or native IL-28B was incubated in human serum with or without protease inhibitor cocktail for 3, 9, or 24 hours at 37°C, and their antiviral activities were determined by measuring intracellular luciferase activity. (B) Pharmacokinetics analysis of HSA-IL28B and native IL-28B. Serum concentrations of fusion proteins at 0, 30 min, 2 h, 6 h, 12 h, 24 h, 48 h, 72 h, 120 h and 144 h post injection were determined by ELISA.

As shown in **Fig. 3A**, HSA-IL28B fusion protein exhibited high stability in human serum. After being incubated for 24 h, the antiviral activity of HSA-IL28B was still above 60% versus 10% for native IL-28B. Moreover, in the presence of a protease inhibitor cocktail, both HSA-IL28B and native IL-28B can maintain the antiviral activity throughout the time course. These results demonstrated that HSA-IL28B fusion protein is more resistant to degradation by proteases than native IL28B protein.

### Pharmacokinetic Analysis

Mean concentration-time profiles of HSA-IL28B and native IL-28B were presented in **Fig. 3B**. After subcutaneous administration, both HSA-IL28B and native IL-28B were rapidly absorbed. After absorption, HSA-IL28B displayed a slow elimination phase with a half-life of about 48 h versus 15 h for native IL-28B.

### HSA-IL28B Inhibits HCV

**Figure 4:**
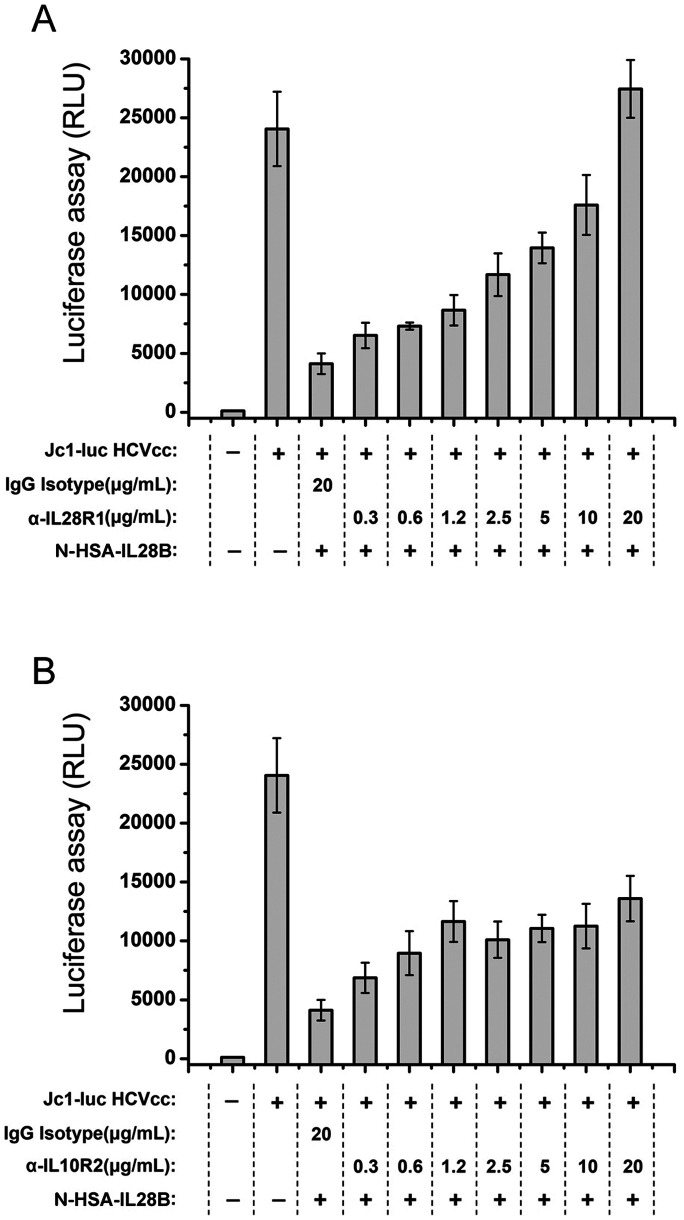
HSA-IL28B acts through IL28R1 and IL10R2. Huh7.5.1 cells were infected with Jc1-Luc HCVcc (multiplicity of infection  = 0.1) 1 day before antibody neutralization and N-HSA-IL28B treatment. Anti-IL28R1 blocking antibody (A) or anti-IL10R2 blocking antibody (B) was added to infected cells and incubated for 2 h before washing. After receptor blocking, cells were further treated with 12 ng/mL N-HSA-IL28B for another 2 days. IgG isotype (20 µg/mL) was used as the negative control. Luciferase assays were performed to measure the propagation of HCVcc.

As shown in **Figs. 4A and 4B**, as the concentration of neutralizing antibodies against IL28R1 or IL10R2 increased, the antiviral effects of HSA-IL28B were gradually reduced. When the concentration of antibody against IL28R1 was 20 µg/mL, HSA-IL28B activity was completely blocked. The antibody against IL10R2 was less potent. The control, isotype IgG, failed to block the antiviral effects of HSA-IL28B (**Fig. 4**). These data demonstrate that HSA-IL28B inhibits HCV replication in an IL28R1- and IL10R2-dependent manner.

### HSA-IL28B Phosphorylates STAT1 and Induces Selected IFN-stimulated Genes (ISGs)

**Figure 5:**
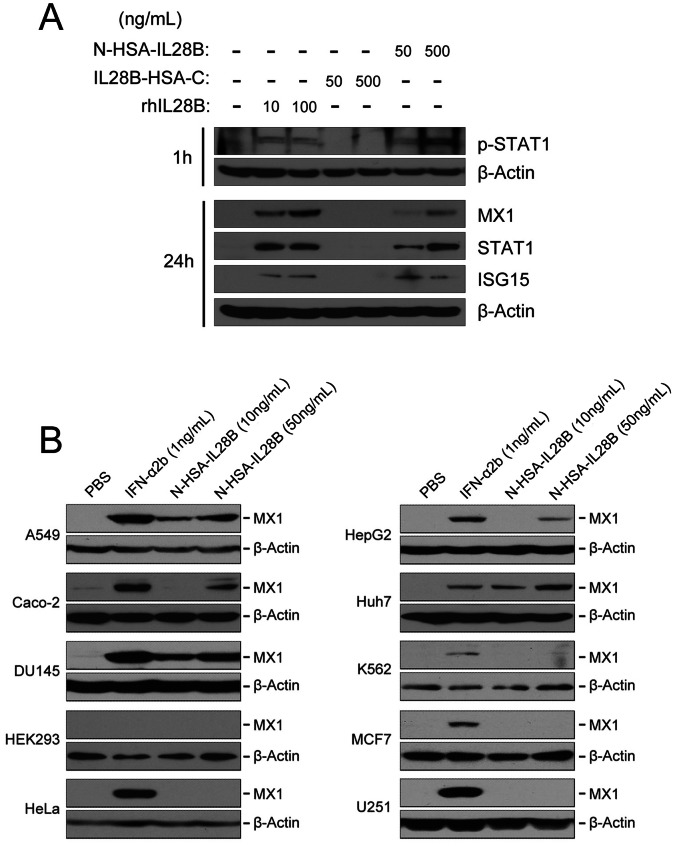
HSA-IL28B stimulates ISGs and functions in restricted cell types. (A) HepG2 cells were treated with N-HSA-IL28B, IL28B-HSA-C, or native IL-28B, as indicated, for 1 or 24 h. Western blots were performed to analyze the indicated proteins. The loading control was β-Actin. (B) A panel of cells derived from multiple tissue origins were stimulated with either N-HSA-IL28B (10 or 50 ng/mL) or IFN-α2b (1 ng/mL) for 24 h before lysing. MX1 expression was detected by western blotting with β-Actin serving as the loading control.

Compared with the untreated control, the phosphorylation of STAT1 occurred within 1 h after N-HSA-IL28B or native IL28B stimulation. IL28B-HSA-C treatment had no effect on STAT1 phosphorylation (**Fig. 5A**). Consistent with STAT1 phosphorylation, a similar induction pattern of MX1, STAT1 and ISG15 by various forms of recombinant IL-28B was also detected at 24 h post-treatment (**Fig. 5A**).

### HSA-IL28B has a More Restricted Cell-type Response than Type I IFNs

IFN-α2b induced MX1 expression in nearly all tested cell types, except for HEK293 at a concentration of 1 ng/mL (**Fig. 5B**). A low dose of HSA-IL28B (10 ng/mL) demonstrated comparable activity to IFN-α2b, but only in Huh7 cells. DU145, A549, HepG2 and Caco-2 cells showed responsiveness to relatively high concentrations of HSA-IL28B (50 ng/mL). HEK293, HeLa, K562, MCF7 and U251 cells showed no responsiveness to both low and high concentrations of HSA-IL28B (**Fig. 5B**). These data imply that albumin fusion IL28B has relatively specific preference to targeting liver, prostate, lung and colonic/gut epithelial cells.

## Discussion

Host IFNs are the first line of defense against viral pathogens. They have inhibitory effects on viral RNA transcription and protein synthesis, either directly or indirectly, through the activation of a panel of ISGs. Although type I IFNs are currently the major therapeutics used in the treatment of hepatitis C, the relatively recent discovery of type III IFNs has opened up new avenues for research and drug development. Importantly, the finding that genetic variation in the IL28B gene is associated with responsiveness to chronic hepatitis C strongly implies that there are biological connections between IL28B and hepatitis C. However, like many low molecular weight polypeptide drugs, the native IL28B is rapidly cleared through the kidney and has a short plasma half-life, reducing its efficacy and bioavailability.

HSA is an ideal candidate for integration into a drug-design platform. Although the *in vitro* activity usually decreased due to the steric hindrance effect of HSA, rHSA-fusion protein exhibited an extended half-life. Thus, its biological activity even increased *in vivo* [14], [18] Preclinical and clinical trials have confirmed the safety and efficacy of using a recombinant HSA (rHSA) preparation for different disease conditions, such as hemorrhagic shock, cirrhosis with ascites, and other critical clinical conditions related to plasma volume and oncotic pressure. In addition to its use as a plasma expander, rHSA has great potential as a biomaterial for other medical and pharmaceutical-related applications [19]. In the last few years, clinical studies showed that IFN-α2b and HSA fusion protein may be as safe and efficacious as pegylated interferon-α2a but require fewer injections and be better tolerated. Unfortunately, the Phase III trials of IFN-α2b and HSA fusion protein were halted due to severe adverse effects [20], [21]. However, as a type III IFN, pegylated IL-29 has been reported with better tolerance and lower adverse effects than pegylated IFN-α [6]. Our previously studies also showed that IL28B has potent antiviral activity against HCV and restricted cell-type responsiveness *in vitro* [15]. Thus, development of longer-acting IL28B is a promising option for the treatment of HCV. In this study, we tried to increase the stability of native IL-28B through covalent linkage to HSA, which we also hoped would reduce the renal clearance rate of IL-28B *in vivo*.

Along with establishing successful production of HSA-IL28B in yeast cells, our data also showed clear evidence that HSA-IL28B has comparable biological activities with the native form of IL-28B. However, our HAS-IL28B has prolonged protein stability *in vitro*. In conclusion, our fusion protein is predicted to have significantly wider therapeutic applications than originally envisaged. Further investigations relating to *in vivo* metabolism and toxicity are currently underway.

## Supporting Information

Figure S1 **MALDI-TOF mass spectrometry analyses of purified HSA-IL28B.**
(TIF)

Figure S2 **Peptide sequences from the human serum albumin (HSA) were identified by mass spectrometry (red).**
(TIF)

Figure S3 **Peptide sequences from the human IL28B were identified by mass spectrometry (red).**
(TIF)
