# Supplementary figures and images for: Albumin Fusion of Interleukin-28B: Production and Characterization of Its Biological Activities and Protein Stability
Source: PLoS One. 2013 May 31;8(5):e64301. doi: 10.1371/journal.pone.0064301 (PMC3669341; doi:10.1371/journal.pone.0064301)

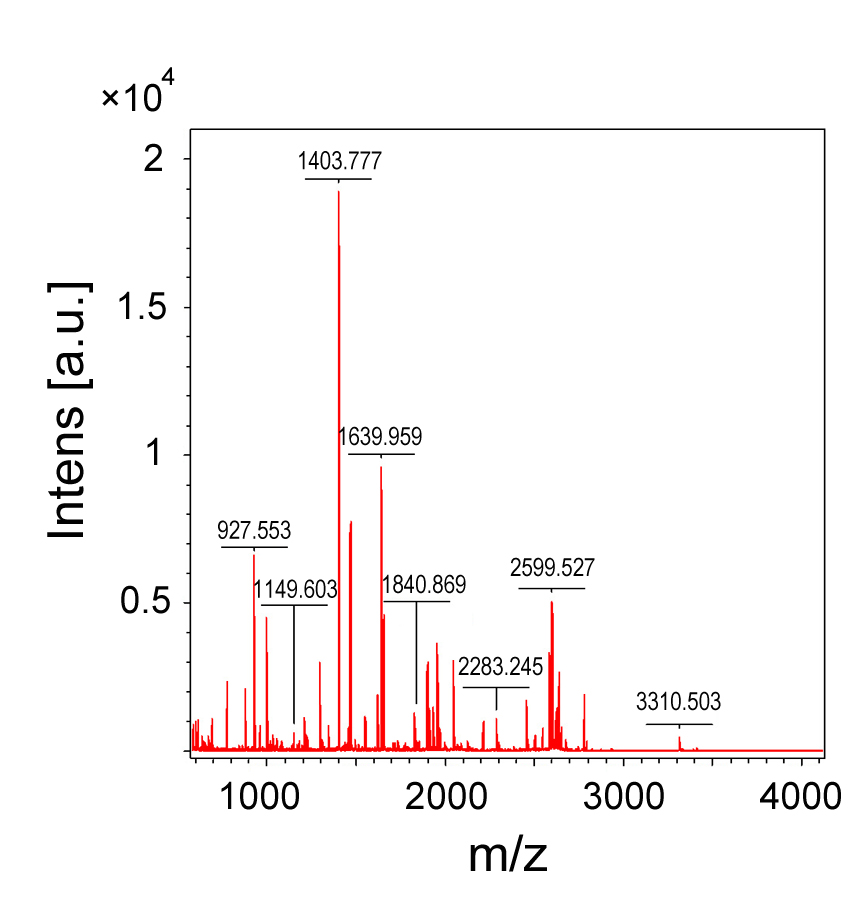

Supplement: Figure S1 — MALDI-TOF mass spectrometry analyses of purified HSA-IL28B. (TIF) [file pone.0064301.s001.tif]

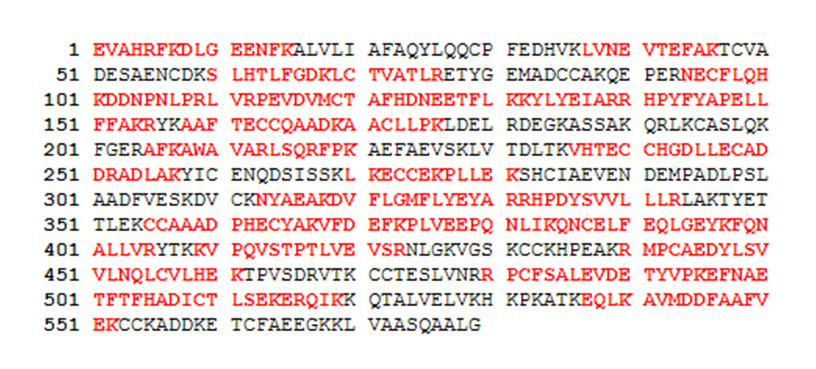

Supplement: Figure S2 — Peptide sequences from the human serum albumin (HSA) were identified by mass spectrometry (red). (TIF) [file pone.0064301.s002.tif]

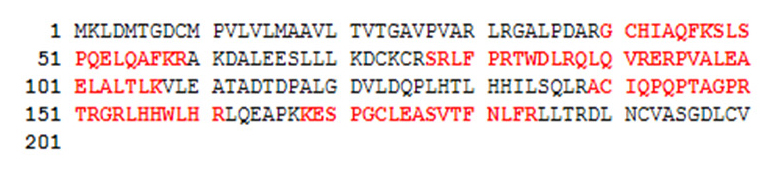

Supplement: Figure S3 — Peptide sequences from the human IL28B were identified by mass spectrometry (red). (TIF) [file pone.0064301.s003.tif]
